# Metabolic gene expression profile in circulating mononuclear cells reflects obesity-associated metabolic inflexibility

**DOI:** 10.1186/s12986-016-0135-5

**Published:** 2016-10-27

**Authors:** Sonia Baig, Ehsan Parvaresh Rizi, Muhammad Shabeer, Vanna Chhay, Shao Feng Mok, Tze Ping Loh, Faidon Magkos, Antonio Vidal-Puig, E. Shyong Tai, Chin Meng Khoo, Sue-Anne Toh

**Affiliations:** 1 Department of Medicine, Yong Loo Lin School of Medicine, National University of Singapore, 14 Medical Drive, Singapore, 117599 Singapore; 2 Department of Medicine, National University Health System, Singapore, Singapore; 3 Department of Laboratory Medicine, National University Health System, Singapore, Singapore; 4 Department of Physiology, Yong Loo Lin School of Medicine, National University of Singapore, Singapore, Singapore; 5 Singapore Institute of Clinical Sciences (SICS), A*STAR, Singapore, Singapore; 6 University of Cambridge Metabolic Research Laboratories, Cambridge, UK; 7 Duke-National University of Singapore Medical School, Singapore, Singapore; 8 Perelman School of Medicine, University of Pennsylvania, Philadelphia, USA

**Keywords:** Mononuclear cells, Metabolic flexibility, Gene expression, Macronutrients, Obesity

## Abstract

**Background:**

Obesity is associated with an impaired ability to switch from fatty acid to glucose oxidation during the fasted to fed transition, particularly in skeletal muscle. However, whether such metabolic inflexibility is reflected at the gene transcription level in circulatory mononuclear cells (MNC) is not known.

**Methods:**

The whole-body respiratory quotient (RQ) and transcriptional regulation of genes involved in carbohydrate and lipid metabolism in MNC were measured during fasting and in response (up to 6 h) to high-carbohydrate and high-fat meals in nine lean insulin-sensitive and nine obese insulin-resistant men.

**Results:**

Compared to lean subjects, obese subjects had an impaired ability to increase RQ and switch from fatty acid to glucose oxidation following the high-carbohydrate meal (interaction term *P* < 0.05). This was accompanied by an impaired induction of genes involved in oxidative metabolism of glucose in MNC, such as phosphofructokinase (PFK), pyruvate dehydrogenase kinase 4 (PDK4), peroxisome proliferator-activated receptor alpha (PPARα) and uncoupling protein 3 (UCP3) and increased expression of genes involved in fatty acid metabolism, such as fatty acid translocase (FAT/CD36) and fatty acid synthase (FASN) (*P* < 0.05). On the contrary, there were no differences in the gene expression profiles between lean and obese subjects following the high-fat meal.

**Conclusions:**

Postprandial expression profiles of genes involved in glucose and fatty acid metabolism in the MNC reflect the differing metabolic flexibility phenotypes of our cohort of lean and obese individuals. These differences in metabolic flexibility between the lean and obese are elicited by an acute meal challenge that is rich in carbohydrate but not fat.

**Electronic supplementary material:**

The online version of this article (doi:10.1186/s12986-016-0135-5) contains supplementary material, which is available to authorized users.

## Background

Normal energy metabolism is characterized by periodic shifts between glucose and fatty acid oxidation according to physiological and nutritional status [1, 2]. Impaired ability for such a switch in fuel oxidation, known as metabolic inflexibility, has been shown to occur early in the pathogenesis of insulin resistance, and is closely associated with obesity [3–5]. Increasingly, obesity-related cardiometabolic complications are recognized as disorders linked to metabolic inflexibility [6–11]. Current evidence supports the notion that metabolic flexibility is indeed observed at the level of gene and protein expression in skeletal muscle tissue samples, obtained by biopsies [12]. Moreover, physiological induction or suppression of a wide range of transcripts, which occurs during the fasting-to-fed transition, is attenuated in models of metabolic disease [13–15].

Circulating mononuclear cells (MNC) can be obtained in large quantities through routine blood draws, in contrast to skeletal muscle biopsy which is invasive and could pose higher risk to the study subjects. A comparison of MNC transcriptome and that of different human tissues demonstrated that over 80 % of gene expression is shared and that these gene transcripts in MNC display changes in the cellular environment [16]. It has been demonstrated that gene transcripts in MNC can provide an accurate representation of transcriptional responses of metabolic tissues (i.e. hepatocytes and adipocytes) to nutrient intake in humans [17–19]. MNCs have been suggested as an alternative to muscle biopsies to study mitochondrial dysfunction [20, 21].

In this study, we examined the transcriptional responses of genes involved in glycolytic, oxidative and fatty acid metabolism in MNC in response to a single high-carbohydrate or high-fat meal among individuals with different metabolic flexibility. We hypothesized that (1) acute meal challenges of differing composition (high carbohydrate vs high fat) would have a different propensity to elicit differences in metabolic flexibility between the lean and obese; and (2) dynamic changes in metabolic gene expression profiles in MNC could reflect the underlying metabolic flexibility status of individuals.

## Methods

### Study approval and subjects

This study was approved by the Singapore’s National Healthcare Group Domain Specific Review Board (DSRB Ref No: C/2013/00902), and all procedures followed the Singapore Good Clinical Practice guideline and the principles of the 2013 Declaration of Helsinki. All subjects provided written consent before participation in this study. We recruited a total of 18 normoglycemic (fasting blood glucose <5.6 mmol/l, no history of diabetes) Chinese men aged 21–40 years. We excluded subjects who are currently smoking, with previous or current thyroid disorder, history of malignancy, hospitalization or surgery during the past 6 months, on treatment for dyslipidemia, use of corticosteroids during the past 3 months, or consume alcohol >3 units daily, perform moderate to high intensity physical activity >5 h per week, or had ≥ 5 % change in their weight within the past 3 months prior to the study. We also excluded those who have a first degree relative with Type 2 diabetes. We used the WHO definition for obesity in Asians to define our lean (18.5 ≤ BMI ≤23 kg/m^2^) and obese (BMI ≥27.5 kg/m^2^) subjects [22]. A Homeostatic Model Assessment-Insulin Resistance (HOMA-IR) score of <1.2 mmol/l.mU/L was used to identify insulin-sensitive lean subjects, and ≥ 2.5 mmol/L.mU/L to identify insulin-resistant obese subjects.

### Experimental design

During the screening visit, subjects’ height, weight, and waist circumference were measured. Fasting blood was collected to measure serum insulin, glucose, electrolytes, and non-esterified fatty acid (NEFA) concentrations, and lipid profile. Subsequently, eligible subjects were invited to study visits at the Investigational Medicine Unit (IMU) on two separate occasions, seven days apart. Subjects arrived at the IMU at 7:30 AM, after a 10-h overnight fast. All subjects were advised to refrain from intensive physical activity for 24 h before each visit. An isocaloric (~600 kcal), isovolumic (~400 ml) liquid mixed meal, rich in either carbohydrate or fat, was given to the subjects in random order and to be ingested within 5 min. The high-carbohydrate and high-fat meals provided ~56 % of total energy from carbohydrate or fat (with a 1:1:1 ratio of saturated, mono- and poly-unsaturated fatty acids; Additional file 1: Table S1), respectively. Fasting and postprandial (30, 60, 90, 120, 180, 240, 300, and 360 min) venous blood samples were collected through an intravenous catheter for the measurement of glucose, insulin, triglycerides and NEFA concentrations. Oxygen consumption (VO_2_) and carbon dioxide production (VCO_2_) were measured through an open-circuit indirect calorimetry system using a ventilated hood (Quark CPET, COSMED®, Italy) for 30 min before the meal and continuously after the meal for 6 h. Respiratory quotient (RQ) was calculated from VO_2_ and VCO_2_ as follows: RQ = VCO_2_/VO_2_.

### Biochemical analysis

Plasma glucose and triglyceride concentrations were measured by using enzymatic and colorimetric methods, respectively (AU5800, Beckman Coulter Inc., California, USA). Serum insulin was measured by using a chemiluminescence immunoassay (ADVIA Centaur, Siemens Healthcare Diagnostics, Hamburg, Germany). These analyses were carried out by a laboratory accredited by the College of American Pathologists. Plasma NEFA was measured at Mayo Medical Laboratories (Rochester, MN, USA), using an enzymatic colorimetric method (Cobas® 6000, Roche Diagnostics, Indianapolis, USA).

### Gene expression

A 9 ml blood sample was collected into tubes containing EDTA at 0, 120, and 360 min. The blood sample was then layered over 9 ml of Ficoll-paque Plus (GE Healthcare, Buckinghamshire, UK) and centrifuged. Phosphate-buffered saline was used to wash the harvested MNC, followed by red blood cell lysis (Sigma-Aldrich, St. Louis, MO, USA). RNeasy Mini Kit (QIAGEN, Netherlands) was used to isolate total RNA from MNC. High capacity cDNA Reverse-Transcription Kit (Applied Biosystems, Waltham, MA, USA) was used to reverse transcribe 500 ng of total RNA. Real-time reverse transcription–polymerase chain reaction (RT-PCR) was performed using ViiA 7 Real-Time PCR System (Applied Biosystems). The PCR mix consisted of 2 μL (10 ng) cDNA, 5 μL QuantiFast SYBR Green PCR Master mix (QIAGEN), and 0.1 μL of 100 μmol/L gene-specific primers (AIT Biotech, Singapore). All the procedures above were performed according to manufacturer’s instructions. Available sequences in the NCBI nucleotide database were used to select primer sequences, using Primer Express software v3.0.1 (Applied Biosystems) (Additional file 2: Table S2). At the end of the amplification, melt curves were analysed to test the specificity of the PCR products. All samples were run in duplicates and variations in the threshold cycle (CT) between technical replicates were within 10 %. All values were normalized to the expression of a housekeeping gene (GAPDH). The expression of GAPDH gene was stable and did not show significant variation across the different time points, meals, and subject’s phenotype. We determined the ability to switch from fatty acid to glucose oxidation in MNC using a panel of genes involved in the regulation of glucose and fatty acid metabolism i.e. hexokinase (HK), phosphofructokinase (PFK), pyruvate dehydrogenase kinase 4 (PDK4), peroxisome proliferator-activated receptor alpha (PPARα), uncoupling protein 3 (UCP3), fatty acid translocase (FAT/CD36), acetyl Co-A carboxylase 2 (ACC2), fatty acid synthase (FASN), stearoyl Co-A desaturase (SCD), Carnitine palmitoyltransferase 1B (CPT1B), and sterol regulatory element -binding protein 1 (SREBP1). Three sets of samples following the high-carbohydrate meal (2 sets from lean subjects, 1 set from obese subjects) were excluded due to insufficient or poor quality of RNA.

### Statistical analysis

A sample size of nine subjects in each group was calculated to provide 80 % power at 5 % significance level to detect differences between the groups in RQ changes from fasting to insulin stimulated (postprandial) state following a meal challenge, as reported previously [3]. Data are shown as means ± SEM unless otherwise stated. Analyses were carried out with SPSS version 23.0 for Windows (SPSS Inc., Chicago, IL, USA). Fasting gene expression levels before ingesting the two experimental meals were tested by paired sample *t-*tests within each group of subjects, to verify there were no differences at baseline between two visits. Differences for averaged values for each group were tested by independent sample *t*-test. The incremental area under the curve (iAUC) was computed using the trapezoidal method. Repeated-measures ANOVA was used to evaluate differences in the RQ esponse to the meal by group and time. We performed a linear mixed model to analyse MNC gene expression differences between groups by meals. Fold-change from baseline in MNC gene expression of interest was entered as the dependent variable. Time and group were entered as factors in the model. We introduced interaction term time × subjects’ group to examine whether the postprandial responses for the gene of interest differed between subjects’ groups. Differences in fold-changes in MNC gene expression at a single time point (120 min or 360 min) between the two groups were tested by independent sample *t*-test. Statistical significance was set at *P* < 0.05.

## Results

### Baseline characteristics

Obese subjects were older, and had greater waist circumference, HOMA-IR, fasting plasma insulin and triglyceride concentrations compared to lean subjects. In contrast, lean subjects had higher fasting HDL-cholesterol concentration. Fasting glucose, total and LDL-cholesterol, and NEFA concentrations were not different between lean and obese subjects (Table 1).

**Table 1:** Baseline characteristics of study participants after a 10-h overnight fast

	Lean subjects	Obese subjects	*P*	*Adjusted P* *
Age (years)	23.2 ± 0.2	28.6 ± 1.4	0.002	
BMI (kg/m^2^)	22.0 ± 0.2	30.2 ± 0.8	<0.001	<0.001
Waist Circumference (cm)	79.9 ± 0.5	100.8 ± 1.0	<0.001	<0.001
Fasting Blood Glucose (mmol/l)	4.33 ± 0.05	4.68 ± 0.12	0.026	0.459
Fasting Serum Insulin (mU/l)	4.31 ± 0.52	21.04 ± 2.27	<0.001	<0.001
Fasting Total Cholesterol (mmol/l)	5.09 ± 0.27	5.28 ± 0.40	0.697	0.791
Fasting LDL-Cholesterol (mmol/l)	3.09 ± 0.29	3.20 ± 0.35	0.823	0.730
Fasting HDL-Cholesterol (mmol/l)	1.71 ± 0.08	1.19 ± 0.06	0.001	0.005
Fasting Triglyceride (mmol/l)	0.62 ± 0.07	1.98 ± 0.24	<0.001	0.007
Fasting NEFA (mmol/l)^a^	0.41 ± 0.04	0.49 ± 0.03	0.037	0.392
HOMA-IR	0.83 ± 0.10	4.34 ± 0.41	<0.001	<0.001
Fasting RQ	0.79 ± 0.01	0.78 ± 0.01	0.784	0.463

Data are presented as the means ± SEM; * age-adjusted, ^a^log transformed comparison of the mean; *BMI* body mass index, *LDL* low-density lipoprotein, *HDL* high-density lipoprotein, *HOMA-IR* homeostasis model assessment for insulin resistance, *NEFA* nonesterified fatty acids, *RQ* respiratory quotient

Fasting expression levels of all investigated genes were averaged, and we observed no significant variation in the fasting expression of all genes between visits. The fasting expression levels of all genes examined, except PDK4, trended lower in obese compared to lean subjects but reached statistical significance only for HK, PFK, PPARα, FASN, and SCD (Additional file 3: Figure S1). The fasting expression level of PDK4 trended higher in obese subjects compared to lean subjects although it did not reach statistical significance.

### Glucose, insulin, triglycerides and NEFA trajectories following meals

Overall, the postprandial responses for insulin and triglycerides were greater in obese than lean subjects (Table 2) while there were no differences between obese and lean subjects for the postprandial glucose and NEFA responses. Table 2 shows the iAUC of plasma glucose, insulin, triglycerides, and NEFA in response to both meals. In obese subjects, the iAUCs for insulin and triglycerides were greater compared to lean subjects in response to both high-carbohydrate (*P* < 0.001 and *P* = 0.067, respectively) and high-fat (*P* = 0.019 and *P* = 0.004, respectively) meals. No significant differences between lean and obese subjects were found in the iAUCs for glucose and NEFA in response to either meal challenge.

**Table 2:** Postprandial serum insulin, glucose, triglyceride, and NEFA responses to the test meals

		HC meal	*P*	HF meal	*P*
Insulin iAUC_0-360 min_ (mU. min. L^-1^)	Obese	20540 ± 2575	0.001	10508 ± 1846	0.019
	Lean	8374 ± 867		5378 ± 703	
Glucose iAUC_0-360 min_ (mmol. min. L^-1^)	Obese	257 ± 40	0.376	147 ± 9	0.685
	Lean	207 ± 37		161 ± 32	
Triglyceride iAUC_0-360 min_ (mmol. min. L^-1^)	Obese	204.0 ± 59.3	0.067	373.3 ± 77.7	0.004
	Lean	79.0 ± 23.0		104.9 ± 16.2	
NEFA iAUC_0-360 min_ (mmol. min. L^-1^)	Obese	-59.33 ± 13.66	0.969	-28.90 ± 20.27	0.874
	Lean	-58.40 ± 19.26		-33.30 ± 18.32	

Data are presented as the mean incremental area under the curve ± SEM; *n* = 9 for each group. *HC* high-carbohydrate, *HF* high-fat; Statistical differences are based on repeated-measures ANOVA

### Changes in RQ following meals

**Fig. 1:**
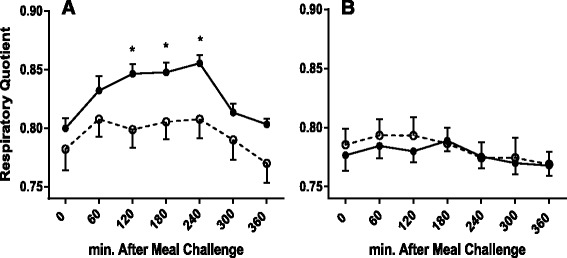
Postprandial changes in respiratory quotient between lean and obese subjects by meals. **a** high-carbohydrate meal (*0–240 min: P* group = 0.045, time <0.001 and time × group = 0.047; *240–360 min: P* group = 0.053, time <0.001 and time × group = 0.099); **b** high-fat meal (*0–240 min*: *P* group = 0.734, time = 0.050 and time × group = 0.482; *240–360 min*: *P* group = 0.927, time = 0.293 and time × group = 0.802). Lean —, ●; obese ----, ○; * *P* < 0.05

There was no statistical difference for the fasting RQ between lean and obese subjects (Fig. 1). The postprandial RQ changes were significant after a high-carbohydrate meal in both lean and obese subjects (time effect *P* < 0.001), but the mean postprandial RQ from 0 to 240 min during the postprandial period was significantly higher in the lean compared to obese subjects (group effect *P* < 0.05). The 0–240 min postprandial RQ trajectories were significantly different between lean and obese subjects (interaction term time x group *P* < 0.05).

For the high-fat meal, there were no statistical differences in the mean postprandial RQ change throughout the postprandial period between lean and obese subjects.

### Changes in metabolic gene expression profiles in MNCs following acute meal challenges

**Fig. 2:**
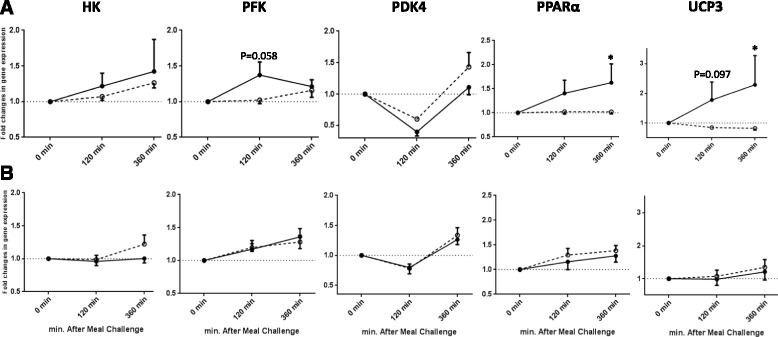
Fold changes from baseline in the expression of genes involved in glycolytic pathway and oxidative metabolism in MNC after ingesting a (**a**) high-carbohydrate or a (**b**) high-fat meal between lean and obese subjects. Significant differential responses, as analysed by linear mixed model, were found after high-carbohydrate meal in expression of: PFK (*P* group = 0.032, and time × group =0.032), PDK4 (*P* group = 0.002, and time × group =0.002), PPARα (*P* group =0.024, and time × group =0.014), and UCP3 (*P* group =0.025, and time × group =0.012). Significant differences at a single time point (120 min or 360 min) between the two groups, as tested by independent sample *t*-test, have been presented in the figure. Data presented as mean ± SEM. Lean —, ●; obese ----, ○; * *P* < 0.05

**Fig. 3:**
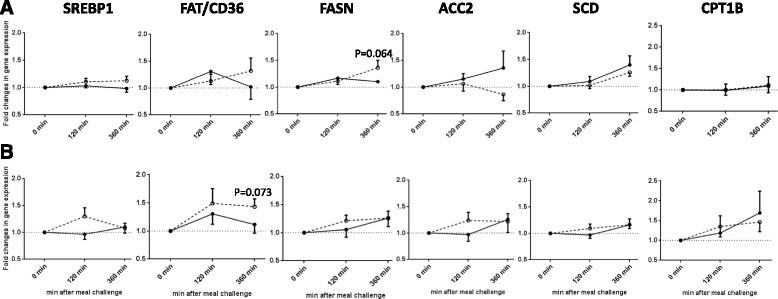
Fold changes from baseline in expression of genes involved in fatty acid uptake and metabolism in MNC after ingesting a (**a**) high-carbohydrate or a (**b**) high-fat meal between lean and obese subjects. Significant differential responses, as analysed by linear mixed model, were found after high-carbohydrate meal in expression of: FAT/CD36 (*P* group = 0.040 and time × group =0.040), FASN (*P* group = 0.083, and time × group =0.083), and ACC2 (*P* group = 0.033, and time × group =0.033). Significant differences at a single time point (120 min or 360 min) between the two groups, as tested by independent sample *t*-test, have been presented in the figure. Data presented as mean ± SEM. Lean —, ●; obese ----, ○; * *P* < 0.05

In response to the high-carbohydrate meal, mean postprandial fold changes for expression of PFK, PDK4, PPARα, UCP3 (Fig. 2a), and ACC2 (Fig. 3a) were significantly different between groups (*P* < 0.05). Obese subjects had a lower increase in PFK (at 2 h), PPARα, UCP3, and ACC2 (at 6 h), and a lower decrease in PDK4 (at 2 h) than lean subjects (interaction term *P* < 0.05). In contrast, mean postprandial fold changes for gene expression of FAT/CD36 and FASN at 6 h were higher in obese compared with lean subjects (Fig. 3a).

After the high-fat meal, no significant differences in the metabolic gene expression profiles in MNCs were seen between lean and obese subjects (Figs. 2b and 3b).

## Discussion

A meal rich in carbohydrate, but not fat was associated with a blunted response in switching from fat to glucose oxidation in obese compared with lean individuals, as indicated by the postprandial RQ change data from whole-body indirect calorimetry. This impaired ability to switch substrate metabolism from fasting to fed state in obese subjects was also reflected in the glycolytic, oxidative and lipid metabolism gene expression profiles in circulating MNC. The lean, being more metabolically flexible, exhibited a sharp induction or suppression of a wide range of metabolic gene transcripts during the fasted-to-fed transition in the MNC, but these responses were attenuated in obese-derived MNC.

Skeletal muscle explants have been conventionally used to study metabolic gene transcripts [13, 14, 23–25]. Data from these studies demonstrate perturbation in metabolic gene expressions in skeletal muscle explants derived from individuals with varying degrees of metabolic health [12–14]. For example, skeletal muscle mRNA expression levels of fatty acid metabolism genes at basal state have been shown to reflect metabolic dysregulation associated with insulin resistance [14]. Also, in a study on individuals with or without heredity of type 2 diabetes challenged with a single high-carbohydrate or high-fat meal, skeletal muscle metabolic gene expression profile was shown to correlate well with metabolic flexibility [13]. However, skeletal muscle biopsy is an invasive procedure that can pose significant risk to the study subject. In patients with high risk of infection (for example patients with type 2 diabetes) or bleeding tendency (patients on anticoagulants), skeletal muscle biopsies may not be feasible [26]. Here, we showed that MNC could be used as an alternative to muscle biopsy explants to interrogate dynamic changes in metabolic gene expression profiles.

In this study, we showed in obese-derived MNC, an impaired ability to upregulate the expression of PFK and UCP3 and downregulate the expression of PDK4 in response to a high-carbohydrate meal. PDK4 phosphorylates and inhibits PDK activity, leading to reduced conversion of pyruvate to acetyl-CoA, impaired glucose oxidation and increased fatty acid oxidation, a phenomenon seen in insulin-resistant individuals [27]. Hence, transcriptional changes in these genes might be a reflection of metabolic inflexibility in metabolic tissues. We further observed that fatty acid uptake and synthesis genes (i.e. FAT/CD36 and FASN), but not genes regulating β-oxidation (i.e. PPARα, ACC2, and CPT1B) were upregulated in obese vs. lean subjects following the high-carbohydrate meal. This likely reflects an overall higher propensity for lipogenesis (fatty acid synthesis and uptake) over lipid oxidation in obese subjects. Consistent with our findings, increased expression of these lipogenic genes has also been reported previously in skeletal muscle of insulin-resistant individuals [14, 25].

We also showed that macronutrient composition of a test meal is important when considering a study on metabolic flexibility, whether measured by RQ changes or assessed by examining cellular gene expressions profiles. In this study, we showed that a high-carbohydrate test meal can elicit the metabolic inflexibility phenotype in obese compared to lean subjects. But such metabolic traits would not be demonstrable if we use a high-fat test meal. Consistent with the metabolic trait found in this study, we also found greater pro-inflammatory postprandial responses to a high-carbohydrate, but not to a high-fat meal in obese compared with lean subjects (manuscript under review). Taken together, a single high-carbohydrate meal can elicit more adverse metabolic effects as compared to a high-fat meal (with a balanced proportion of SFA, PUFA, and MUFA) among obese individuals. The cumulative adverse metabolic effects from high-carbohydrate meals may predispose them to higher cardiometabolic disorders in the long term. In a previous report, metabolic inflexibility phenotype in individuals with heredity of type 2 diabetes was shown to be elicited by an acute meal challenge that is rich in fat, but not carbohydrate [13]. Differences in meal composition (76 % of energy from fat or carbohydrate, as opposed to 56 % in our study) and subject characteristics (obese with family history of type 2 diabetes, as opposed to no family history of type 2 diabetes in our study) could explain the discrepant results.

There are several limitations to this study. Despite having a well characterized cohort of lean insulin-sensitive and obese insulin-resistant individuals, our sample size was relatively small. Still, our study was adequately powered to detect significant differences in RQ and transcriptional changes in genes involved in substrate oxidation. We only studied Chinese men, and future study will be required to validate these results in a larger cohort consisting of other ethnic groups as well as in women. Lastly, gene and protein expression profiling in the skeletal muscle biopsies and circulating MNC concurrently derived from the same subjects would be ideal to validate our findings.

## Conclusions

In conclusion, we found that obesity-associated metabolic inflexibility is demonstrable in the circulating MNC at the level of transcription of genes involved in lipid and carbohydrate metabolism. Importantly, adverse metabolic responses were more pronounced following a high-carbohydrate, but not a high-fat acute meal challenge. Our study suggests that macronutrient composition should be taken into consideration in nutritional intervention strategies for obese individuals to reduce weight or to improve metabolic profiles.

## Additional files

Additional file 1: Table S1. Macronutrient composition of liquid mixed meals. (XLSX 9 kb)
Additional file 2: Table S2. Primers used for SYBR Green Assay and their properties. (XLSX 11 kb)
Additional file 3: Figure S1. Expression levels of genes involved in the glycolytic pathway, oxidative metabolism, and fatty acid uptake and metabolism in MNC of obese and lean subjects, in the fasting state (* *P* < 0.05). (PPTX 89 kb)

## Abbreviations

ACC2: Acetyl Co-A carboxylase 2
BMI: Body mass index
CPT1B: Carnitine palmitoyltransferase 1B
FASN: Fatty acid synthase
FAT/CD36: Fatty acid translocase
HDL: High density lipoprotein
HK: Hexokinase
HOMA-IR: Homeostatic model assessment-insulin resistance
iAUC: Incremental area under the curve
LDL: Low density lipoprotein
MNC: Mononuclear cells
NEFA: Non-esterified fatty acid
PDK4: Pyruvate dehydrogenase kinase 4
PFK: Phosphofructokinase
PPARα: Peroxisome proliferator-activated receptor alpha
RQ: Respiratory quotient
RT-PCR: Real-time reverse transcription–polymerase chain reaction
SCD: Stearoyl Co-A desaturase
SEM: Standard error of the mean
SREBP1: Sterol regulatory element -binding protein 1
UCP3: Uncoupling protein 3
WHO: World health organization

## References

[1] Randle PJ (1998). Regulatory interactions between lipids and carbohydrates: the glucose fatty acid cycle after 35 years. Diabetes Metab Rev.

[2] McGarry JD (2002). Banting lecture 2001: dysregulation of fatty acid metabolism in the etiology of type 2 diabetes. Diabetes.

[3] Kelley DE, Goodpaster B, Wing RR, Simoneau JA (1999). Skeletal muscle fatty acid metabolism in association with insulin resistance, obesity, and weight loss. Am J Physiol.

[4] Blaak EE, Hul G, Verdich C, Stich V, Martinez A, Petersen M, Feskens EF, Patel K, Oppert JM, Barbe P (2006). Fat oxidation before and after a high fat load in the obese insulin-resistant state. J Clin Endocrinol Metab.

[5] Prior SJ, Ryan AS, Stevenson TG, Goldberg AP (2014). Metabolic inflexibility during submaximal aerobic exercise is associated with glucose intolerance in obese older adults. Obesity (Silver Spring).

[6] Ukropcova B, Sereda O, de Jonge L, Bogacka I, Nguyen T, Xie H, Bray GA, Smith SR (2007). Family history of diabetes links impaired substrate switching and reduced mitochondrial content in skeletal muscle. Diabetes.

[7] Turer AT, Malloy CR, Newgard CB, Podgoreanu MV (2010). Energetics and metabolism in the failing heart: important but poorly understood. Curr Opin Clin Nutr Metab Care.

[8] Lee S, Rivera-Vega M, Alsayed HM, Boesch C, Libman I (2015). Metabolic inflexibility and insulin resistance in obese adolescents with non-alcoholic fatty liver disease. Pediatr Diabetes.

[9] Di Sarra D, Tosi F, Bonin C, Fiers T, Kaufman JM, Signori C, Zambotti F, Dall’Alda M, Caruso B, Zanolin ME (2013). Metabolic inflexibility is a feature of women with polycystic ovary syndrome and is associated with both insulin resistance and hyperandrogenism. J Clin Endocrinol Metab.

[10] Bergouignan A, Antoun E, Momken I, Schoeller DA, Gauquelin-Koch G, Simon C, Blanc S (2013). Effect of contrasted levels of habitual physical activity on metabolic flexibility. J Appl Physiol (1985).

[11] Muoio DM (2014). Metabolic inflexibility: when mitochondrial indecision leads to metabolic gridlock. Cell.

[12] Pilegaard H, Saltin B, Neufer PD (2003). Effect of short-term fasting and refeeding on transcriptional regulation of metabolic genes in human skeletal muscle. Diabetes.

[13] Heilbronn LK, Gregersen S, Shirkhedkar D, Hu D, Campbell LV (2007). Impaired fat oxidation after a single high-fat meal in insulin-sensitive nondiabetic individuals with a family history of type 2 diabetes. Diabetes.

[14] Jans A, Sparks LM, van Hees AM, Gjelstad IM, Tierney AC, Riserus U, Drevon CA, Roche HM, Schrauwen P, Blaak EE (2011). Transcriptional metabolic inflexibility in skeletal muscle among individuals with increasing insulin resistance. Obesity (Silver Spring).

[15] Gao AW, Canto C, Houtkooper RH (2014). Mitochondrial response to nutrient availability and its role in metabolic disease. EMBO Mol Med.

[16] Liew CC, Ma J, Tang HC, Zheng R, Dempsey AA (2006). The peripheral blood transcriptome dynamically reflects system wide biology: a potential diagnostic tool. J Lab Clin Med.

[17] de Mello VD, Kolehmanien M, Schwab U, Pulkkinen L, Uusitupa M (2012). Gene expression of peripheral blood mononuclear cells as a tool in dietary intervention studies: What do we know so far?. Mol Nutr Food Res.

[18] Bouwens M, Afman LA, Muller M (2007). Fasting induces changes in peripheral blood mononuclear cell gene expression profiles related to increases in fatty acid beta-oxidation: functional role of peroxisome proliferator activated receptor alpha in human peripheral blood mononuclear cells. Am J Clin Nutr.

[19] Boucher P, Seree E, Vidon C, de Souza AC, Villard PH, Chambon R, Barra Y, Vallon JJ (2000). Dietary lipids affect human ethanol-inducible CYP2E1 gene expression in vivo in mononuclear cells. Life Sci.

[20] Marriage BJ, Clandinin MT, MacDonald IM, Glerum DM (2003). The use of lymphocytes to screen for oxidative phosphorylation disorders. Anal Biochem.

[21] Abu-Amero KK, Bosley TM (2005). Detection of mitochondrial respiratory dysfunction in circulating lymphocytes using resazurin. Arch Pathol Lab Med.

[22] Galgani JE, Heilbronn LK, Azuma K, Kelley DE, Albu JB, Pi-Sunyer X, Smith SR, Ravussin E (2008). Metabolic flexibility in response to glucose is not impaired in people with type 2 diabetes after controlling for glucose disposal rate. Diabetes.

[23] Cameron-Smith D, Burke LM, Angus DJ, Tunstall RJ, Cox GR, Bonen A, Hawley JA, Hargreaves M (2003). A short-term, high-fat diet up-regulates lipid metabolism and gene expression in human skeletal muscle. Am J Clin Nutr.

[24] Frier BC, Jacobs RL, Wright DC (2011). Interactions between the consumption of a high-fat diet and fasting in the regulation of fatty acid oxidation enzyme gene expression: an evaluation of potential mechanisms. Am J Physiol Regul Integr Comp Physiol.

[25] Hall LM, Moran CN, Milne GR, Wilson J, MacFarlane NG, Forouhi NG, Hariharan N, Salt IP, Sattar N, Gill JM (2010). Fat oxidation, fitness and skeletal muscle expression of oxidative/lipid metabolism genes in South Asians: implications for insulin resistance?. PLoS One.

[26] Bertoni AG, Saydah S, Brancati FL (2001). Diabetes and the risk of infection-related mortality in the U.S. Diabetes Care.

[27] Majer M, Popov KM, Harris RA, Bogardus C, Prochazka M (1998). Insulin downregulates pyruvate dehydrogenase kinase (PDK) mRNA: potential mechanism contributing to increased lipid oxidation in insulin-resistant subjects. Mol Genet Metab.

