# Supplementary figures and images for: Metabolic gene expression profile in circulating mononuclear cells reflects obesity-associated metabolic inflexibility
Source: Nutr Metab (Lond). 2016 Oct 27;13:74. doi: 10.1186/s12986-016-0135-5 (PMC5081666; doi:10.1186/s12986-016-0135-5)

## Slide 1
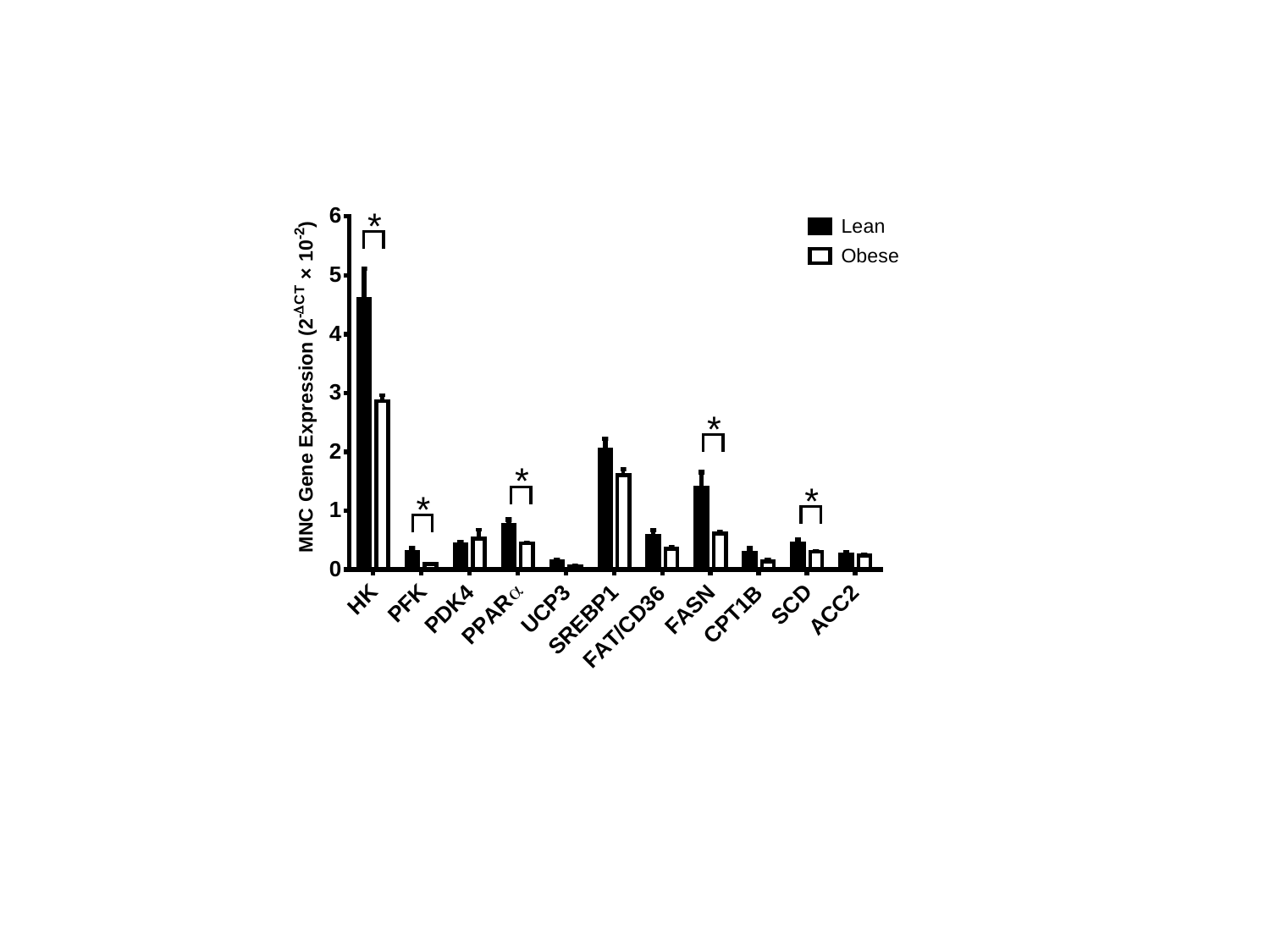

Supplement: Additional file 3: Figure S1. — Expression levels of genes involved in the glycolytic pathway, oxidative metabolism, and fatty acid uptake and metabolism in MNC of obese and lean subjects, in the fasting state (* P < 0.05). (PPTX 89 kb) [file 12986_2016_135_MOESM3_ESM.pptx]
